# Polarised neutron scattering from dynamic polarised nuclei 1972–2022

**DOI:** 10.1140/epje/s10189-023-00295-6

**Published:** 2023-06-06

**Authors:** Heinrich B. Stuhrmann

**Affiliations:** 1 Institut de Biologie Structurale, 38000 Grenoble, France; 2 Helmholtz Zentrum Hereon, 21502 Geesthacht, Germany

## Abstract

With the inauguration of the small-angle instrument D11 of the Institute Laue–Langevin (ILL) in September 1972 neutron scattering revolutionized methods of contrast variation. Very soon D11 was oversubscribed by proposals relying on isotopic substitution of hydrogen isotopes. At the same time in Oxford first experiments of polarised neutron diffraction from dynamic polarised protons in lanthanum magnesium nitrate crystals demonstrated the great utility of this approach. In the early eighties a new type of polarised target material led to a boom of contrast variation by nuclear polarisation. The new samples of frozen solutions of macromolecules lent themselves to small-angle scattering. Often in collaboration with research centres of High Energy Physics various groups in Europe and Japan started experiments of polarized neutron scattering from dynamic polarised protons. Techniques of NMR and EPR considerably enlarged the spectrum of nuclear contrast variation. This is shown with time-resolved polarised neutron scattering from dynamic polarized proton spins of a free radical and of tyrosyl doped catalase using D22 at the ILL.

**Graphical abstract:**

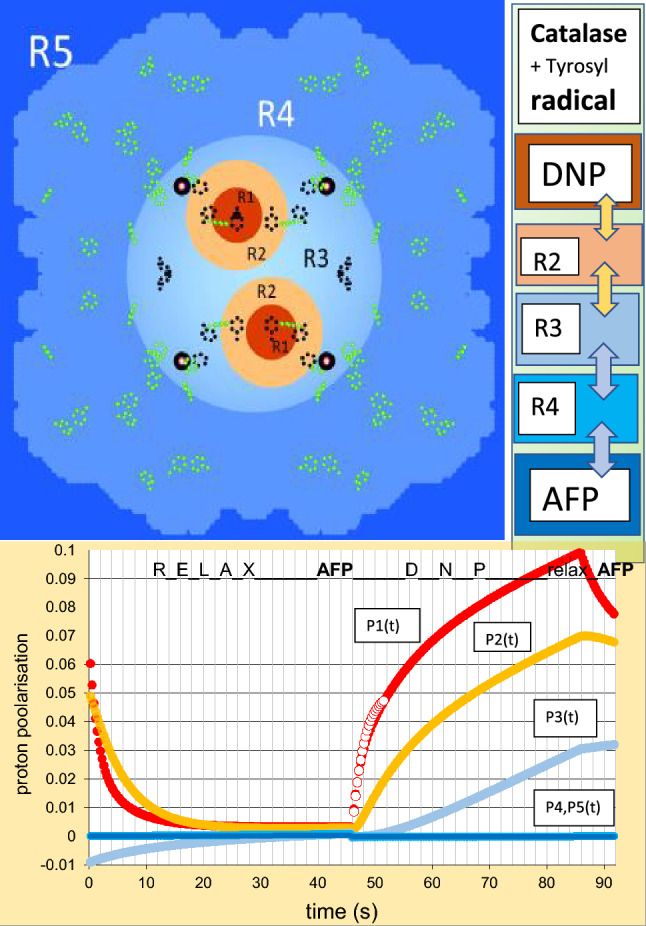

## Sheet lightning

It was in September 1972 when Konrad Ibel and myself put a solution of sperm whale myoglobin into the sample chamber of D11. To our great surprise it was after a few seconds of irradiation by cold neutrons—the A-selector was not yet in place—when a beautiful central peak of scattered neutron intensity emerged on the screen, the first picture of neutron small-angle scattering with D11.

This was also the beginning of neutron small-angle scattering from macromolecules in mixtures of heavy water, D_2_O, and H_2_O. The large difference in the scattering lengths of the isotopes ^1^H (= H) and ^2^H (= D) has been beneficial for studies of composite structures like membranes, nucleoproteins, lipoproteins, and viruses. The demand of beam time largely exceeded the time available at D11.

It was 2 years later in 1974 at the Laboratory of Physical Chemistry Oxford, when Hayter, Jenkins and White [1] came up with a method using the spin dependence of the interaction of polarised neutrons with polarised protons. The variation of the scattering length b with nuclear polarisation is significant for the hydrogen isotopes 1H (= H) and to a lesser extent for 2H (= D). For protons (= H) it largely exceeds that obtained with isotopic substitution.

1 $$ b_{H} = \left( { - 0.374 + p P_{H} 1.456} \right) 10^{ - 12} \,{\text{cm }} $$

2 $$ b_{D} = \left( { + 0.667 + p P_{D} 0.28} \right) 10^{ - 12} \,{\text{cm }} $$

The polarisation of the neutron beam is close to $\left| p \right| = 1$. High nuclear polarisation is achieved by the method of dynamic nuclear polarisation (DNP). Some properties of DNP will be alluded to in the course of this presentation.

**Fig. 1:**
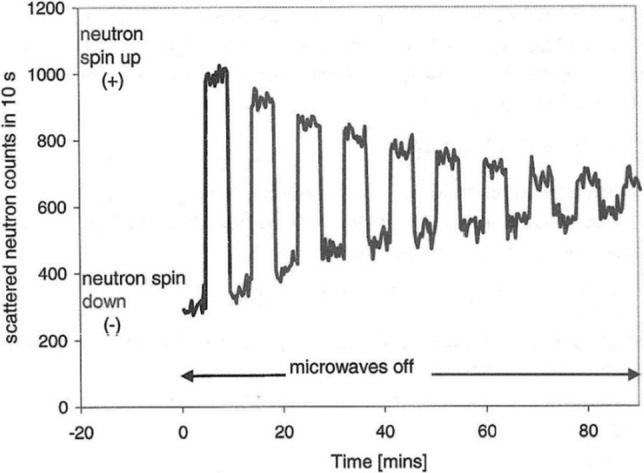
Decay of the flipping ratio and coherent scattering intensity for the (3, 0, 12) Bragg peak after turning off the microwave power. The nuclear relaxation time was 25 min. After Hayter et al. [1]

In 1974, John Hayter, Graham Jenkins and John White [1] reported in their PRL paper on first experiments of polarized neutron diffraction from spin polarised protons in a crystal of lanthanum magnesium nitrate (LMN). The most obvious effect of nuclear spin polarisation was that the cross section of neutron scattering became dependent on the neutron spin direction (Fig. 1). Dynamic polarized nuclear spin targets are surprisingly stable. A nuclear spin relaxation time of 25 s has been observed at T = 1 K (Fig. 1). The authors had hoped from the beginning to use NMR line saturation (as in ENDOR) for the discrimination between isotopes. In a further step the discrimination between nuclei of the same isotope but different Larmor frequency due to the proximity to a radical would have tremendously enlarged the tools of molecular structure research [1].

Later these experiments were continued at the Institute Laue Langevin (ILL). From a limited set of data, the hydrogen positions inside the unit cell of LMN have been determined with a good accuracy [2]. An interesting physical phenomenon discovered was the variation of proton spin polarisation throughout the crystal giving rise to a broad background to the diffraction pattern. This work at the ILL was largely influenced by Graham Jenkin.

In a recent message, John White let me know about the great contributions of John Hayter, who did the first experiments with the Q-band equipment at Harwell, rewrote the Pluto reactor polarised neutron diffractometer software, and collaborated with Oxford Instruments on the superconducting magnet for the 4 mm microwave experiments. Stephen Cox, at that time at the Rutherford Laboratory, in collaboration with the Oxford workshops made the phase-locked NMR and EAR electronics stable to 1 Hz.

In a private communication (letter from 14. December 1983) John Hayter let me know that proton polarisation by brute force would be more promising. There was not much left from the initial hope expressed in 1974. Sheet lightning without thunderstorm?

## A new start

It was in June 1975 when Sir John Kendrew offered me the position of Head of the EMBL Outstation at DESY in Hamburg. I got leave from University of Mainz and I moved with my family to Hamburg. The task at the EMBL Outstation was immense. An empty hall which was expected to receive synchrotron radiation from the positron ring accelerator of DORIS had to be equipped with various kinds of diffractometers and spectrometers considered to be useful in biological structure research. The technical development and the scientific progress entirely depended on the availability of synchrotron radiation, a situation which in no way could be compared to the comfortable one offered to the users of the instruments at the ILL. Thanks to the good contacts with the technical staff of DESY and the comprehension of the Directors of DESY our visiting scientists and those at EMBL achieved a remarkable progress particularly in the field of time-resolved X-ray diffraction.

After my leave from EMBL and return to the university of Mainz I continued my work on Anomalous Small-Angle X-ray scattering (ASAXS) at DESY. Looking for native labels in structural biology I decided to try the anomalous dispersion near the absorption edges of sulphur and phosphorus. In a later stage we used this technique for multi wavelength anomalous diffraction (MAD) from single crystals of proteins and of ribosomes.

It was in summer 1983 when my dream of polarized neutron scattering from dynamically polarised protons became within reach. My proposal had been accepted and funded by the BMFT Bonn. For technical reasons the polarised target station would be installed at the FRG2 reactor of the GKSS Research Centre Geesthacht.

I approached various places in my country asking for help in the construction of a polarised target facility. Oxford Instruments had been contacted as well. Finally, Reinhard Scherm showed me the way to go. It led me to CERN.

It was on a nice Sunday morning, the 23rd December 1984, when I, accompanied by my wife, met Tapio Niinikoski in his office at CERN. In a discussion of about 2 h, I explained to him my intention to polarise the protons of an enzyme in a deuterated glass forming solvent in the presence of EHBA-Cr(V). Tapio assured me that DNP would work. He offered to me a time for a test from 24 February till 10 March 1985.

At the appointed time I came to CERN, this time accompanied by Otto Schärpf. His neutron spin polarisers and spin flipper had just been tested at GKSS (now HZG) a few months before [3, 4]. Thus, an important step towards polarised neutron scattering had already been accomplished. It happened that on the second day at CERN when a friend of mine approached me in the canteen asking what a chemist like me might be looking for at this place. It was Herwig Schopper, whom I knew as Director at DESY during my stay at EMBL and who had become Director General of CERN in the meantime. I explained to him my intention and asked him for an appointment to report at the end of the test experiments. The test was successful. The protons of lysozyme were polarised in less than half hour to 70%. An appointment of 10 min duration was given. With Otto Schärpf as a silent listener I gave a short report that was favourably received by the Director General. This was the birth of a new generation of polarised neutron scattering from dynamic polarised protons in Europe [5].

## New polarised target materials, new horizons

The lysozyme sample mentioned above belongs to the new generation of polarised target materials. Its preparation is simple. The protein is dissolved in a mixture of equal volumes of glycerol and water. A small amount of the very soluble organic complex of chromium(V), C_12_H_20_CrO_7_Na·H_2_O, abbreviated as EHBA-Cr(V), is added. Its synthesis is described by Krumpolc et al. [6]. The solution is deoxygenated and then shock frozen in a copper mould held at liquid nitrogen temperature. The deeply red coloured glass is transferred to the facility of dynamic polarisation.

The glycerol-water mixture can be replaced by any other glass forming solvent. Differential scanning calorimetry (DSC) is recommended to confirm the glass phase. There is a limited choice of radical molecules which support DNP very efficiently. TEMPO belongs to this class. Others will be presented below.

**Fig. 2:**
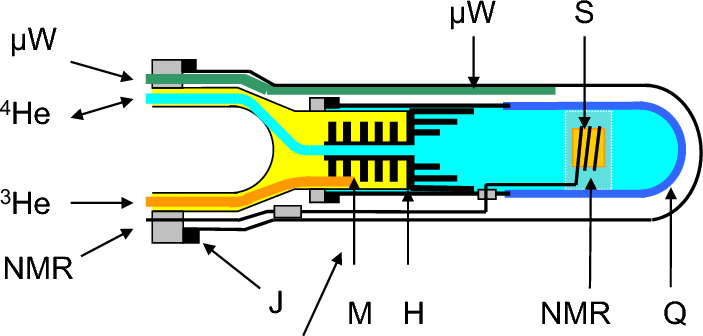
Sample chamber of the dilution refrigerator (designed and constructed by CERN). A quartz cell (Q) filled with liquid helium is connected to the ^3^He/^4^He filled mixing chamber (M) by a heat exchanger (H) made of copper blades covered with sintered copper powder. The NMR coil (NMR) is wrapped around the sample (S) held by a housing made of MACOR (thanks to Jinkui Zhao). The µW guide (µW) may not touch the sample cell. The sample cell and the inner vacuum chamber are sealed by indium joints (J). The axis of the refrigerator is horizontal. The neutron beam is orthogonal to the drawing plane (after Stuhrmann) [8]

The new target materials meant a break-through in all experiments depending on high nuclear polarisation [7]. For the first time a proton polarisation close to 100% could be achieved at temperatures well below 1 K after prolonged microwave irradiation. This was also a challenge for the cryogenics which necessarily relied on dilution refrigerators [7]. While the presence of ^3^He in ^4^He as a coolant is not a problem in many cases, such as in experiments of High Energy Physics, it becomes a real nightmare in neutron scattering. Thermal neutrons are strongly absorbed by ^3^He. The sample chamber shown in Fig. 2 consists of a heat exchanger between the mixing chamber and the sample cell filled with ^4^He transparent to sub-thermal neutrons [8].

**Fig. 3:**
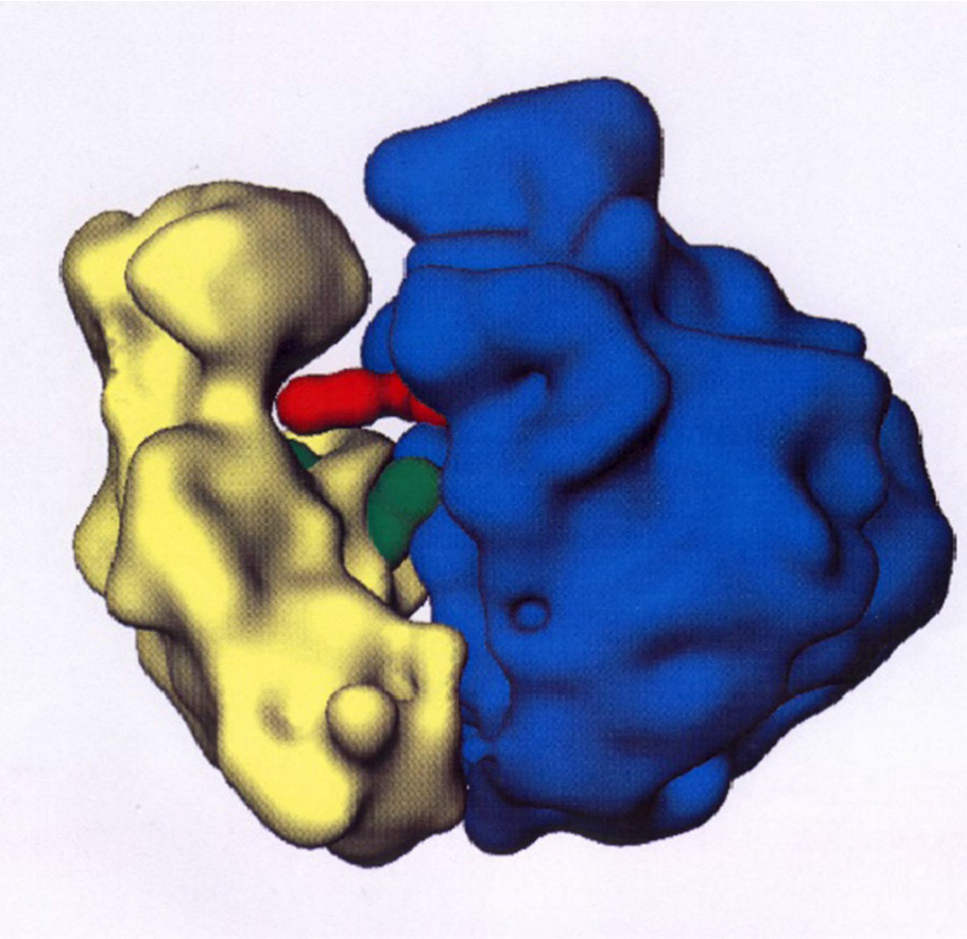
In situ structure of the two tRNA molecules (in red and green, respectively, hydrogen as ^1^H) of the ribosomal functional complex (large subunit in blue, small subunit in yellow, hydrogen largely as ^2^H). Solvent: ^2^H_2_ O/C_3_^2^H_8_ O_3_ in equal volumes. Each tRNA molecule is described by an L-shaped arrangement of four spheres. The anticodon of the tRNA is close to the neck of the small subunit (left) whereas the aminoacyl group approaches the central protuberance of the large subunit (right). The sites of the tRNAs are those which provide the best agreement of the experimental data and the polarisation dependent scattering function of the model (see Eq. 3). The model of the ribosome with its 270 Å diameter has been obtained by electron microscopy [9]. After Nierhaus et al. [10]. The experiment was done at the FRG2 reactor of GKSS at Geesthacht

While the sample chamber shown in Fig. 3 looks complicated, it allows loading of the sample, its study and unloading within 1 day. A single experienced person will do it. Failures have been rare. This refrigerator has been used at the reactor of the GKSS Research Centre for 10 years in the nineties, mainly for studies on the structural studies on ribosomes (Fig. 3). Typically, the sample was loaded on Monday morning. The neutron scattering intensity of the unpolarised sample at T = 0.1 K was measured in the afternoon and then the sample was dynamically polarised for a few hours. Microwaves were switched off. The polarised deuterons were selectively depolarised. The intensity of polarised neutron small-angle scattering of the perfectly stable proton spin target at $P_{H}$ = 0.8 was recorded by an area detector for 2 days. The direction of the neutron polarisation $p$ (see Eq. 3) was changed each 10 min. Then the sample was again dynamically polarised, and the protons were selectively depolarised. The deuteron spin target was studied for another 2 days. Typical polarisation of ^2^H nuclei: around 0.2. The unpolarised protons of the tRNA tend to repolarize. The protons had to be depolarized each quarter of an hour. We recall that the ribosomes dissolved in a mixture of D_2_O and deuterated glycerol were largely deuterated, one or two of its constituents excepted, as it is shown in Fig. 3. The neutron scattering data from the functional ribosome shown in Fig. 3 confirmed the site of the tRNA molecules between the two subunits of the ribosome [9].

The interest in spin dependent neutron scattering amplitudes has been shown by Abragam et al. [11, 12]. In the same year the book of A. Abragam and M. Goldman on *Nuclear Magnetism, Order and Disorder* appeared, with its chapter 7: *Nuclear Magnetism and Neutrons. Nuclear Pseudomagnetism* [12, 13]. Its sub-chapter B on *Slow Neutron Scattering by a (Polarised) Target* contains the master equation, the cross section of coherent scattering given by

3 $$ \left( {\frac{d\sigma }{{d\Omega }}} \right)_{coh.} = \left| {B_{0} } \right|^{2} + \frac{1}{4} \left| B \right|^{2} + p{\text{ Re}}\left( {B_{0} B^{*} } \right) $$

Early experiments of polarised neutron scattering from dynamic polarised targets use the inversion of the polarisation $p$ of the neutron beam [1, 10]. B is the amplitude of polarised nuclei. A more recent presentation of neutron scattering from dynamic polarised targets is due to Glättli and Goldman [14].

Nuclear spin contrast variation has found numerous applications in structural studies on polymers and biomolecules [15–18] and more recently in reflectometry [19–21]. These experiments have in common

1. High proton polarisation: P ≤ 0.8 at T = 0.1 K; P ≤ 0.4 at T = 1 K.
2. Uniform nuclear polarisation throughout the sample volume.

The second hypothesis may be questioned [2].

## A new challenge

The interest in the build-up of proton polarisation emerged from a question concerning the mechanism of bovine liver catalase (BLC). BLC is a redox enzyme which decomposes hydrogen peroxide into water and oxygen at an incredibly fast, diffusion limited rate. By offering peroxyacetic acid, a derivative of hydrogen peroxide with more steric hindrance to bovine liver catalase, the reaction differs in at least two points: first, the response is slow and, second, after some intermediate steps, one of its amino acids, tyrosine, is converted to a tyrosyl radical [22]. Tyrosyl is a native label, as it is part of the enzyme. The number of tyrosyl radicals created in this way is low, typically less than one among the 500 amino acids of one subunit of the catalase molecule [23].

This is the message I got at a symposium on radical proteins held at the Chateau de Sassenage near Grenoble in summer 1997. More precisely, an EPR profile aroused my interest because it looked like that of EHBA-Cr(V) known for its very efficient support of DNP. Could the tyrosyl radical support DNP in the same way? And if so, what could it imply in structural terms? With these ideas in mind, I addressed the speaker, Hélène Marie Jouve, after her lecture, just before lunch, and let her know what was intriguing me. Very much to my surprise she immediately understood and within less than 10 min we agreed on a new way to look at the structure of catalase. This was the starting point of time-resolved polarised neutron scattering in general, and from tyrosyl doped bovine liver catalase in particular.

The idea was that protons near radical sites inside molecular structures might preferentially get polarised and thus rendered visible in experiments of polarised neutron scattering. The special role of protons close to radical sites (also called paramagnetic impurities) has been investigated since the early times of NMR. In 1949 Bloembergen [24] postulated that nuclear spins near a paramagnetic centre are relaxed by fluctuations in the magnetic field of this impurity (direct relaxation) and that bulk spins are relaxed by diffusion of Zeeman energy to near nuclei (nuclear spin diffusion) [25]*.* This observation from NMR is easily translated into a microscopic picture of DNP. Nuclear polarisation occurs near paramagnetic centres through the electron nuclear dipolar interaction decreasing with the third power of the distance between electron and nuclear moments [25]. More distant bulk protons are polarized by dipolar interactions between adjacent nuclei. Fortunately, the potential of this perspective led to a collaboration between French, Swiss and German scientists.

**Fig. 4:**
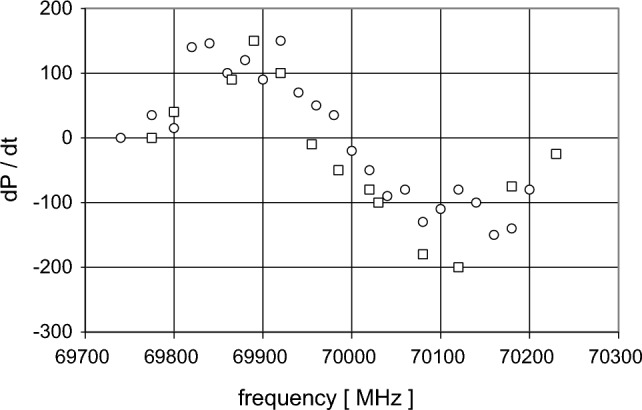
The direction of DNP changes with the microwave frequency. T = 1 K, B = 2.5 T, radical concentration 10^18^ e^−^/cm^3^. The sample is a frozen solution of tyrosyl which has been obtained by irradiating tyrosine with UV light. Courtesy of Jacques Gaillard, CENG. The experiment of DNP was done at the Paul-Scherrer Institute at Villigen, Switzerland

In a first step, we showed that a frozen solution of tyrosyl does support DNP (Fig. 4).

The corresponding result from tyrosyl in catalase was less encouraging. The proton polarisation was disappointingly slow, but it could be inverted by an adiabatic fast passage (AFP) rf sweep over the NMR line which was more impressive [26, 27]. The concentration of radicals was about 20 times lower than with the frozen tyrosyl solution. From this finding it became evident that radical sites surrounded by a flock of polarised protons would show up more clearly with solutions of free radicals present in greater number.

The collaboration of the group of Hans Glättli (CEA, Saclay) focusing on the study of free radical molecules, we are going to discuss now, was therefore extremely welcome.

### Uniform polarisation of solute: two reservoirs ${\varvec{R}}_{1}$ and ${\varvec{R}}_{2}$

**Fig. 5:**
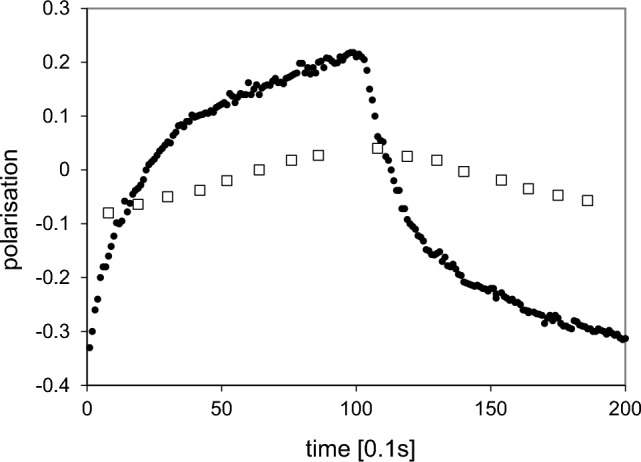
Time-resolved polarised neutron scattering (•••••) and NMR (□ □ □) from dynamic polarised protons of EHBA-Cr(V) in a deuterated solvent. The bis(2-hydroxy-2-ethylbutyrato) oxochromate anion is surrounded by 20 hydrogens of its four ethyl groups [6]. Their polarisation gives rise to an impressive change of the neutron scattering intensity after each change of the direction of DNP. The direction of polarisation was changed every 10 s. The delayed polarisation of the remote protons of the deuterated solvent seen by NMR proceeds more slowly (after van den Brandt) [28]. The experiment was done at D22 in the ILL

The polarised close protons, typically those of a free radical molecule of small size defining $R_{1}$, are embedded in a deuterated solvent containing very few residual protons which constitute $R_{2}$. Examples are the solutions of tyrosyl radical (Fig. 4), and EHBA-Cr(V) mentioned above. Figure 5, for example, shows the evolution of time resolved polarised neutron scattering and of NMR from EHBA-Cr(V) in a deuterated solvent using alternating directions of DNP [28]. Proton polarisation proceeds in two steps: close protons, i. e. those of the four ethyl groups of the EHBA-Cr(V) molecule [6] are polarised within less than one second as it can be seen from the change of the neutron scattering intensity after each change of DNP [28]. More remote protons of the deuterated solvent are polarised by nuclear spin diffusion. These are seen by NMR (Fig. 5).

One might argue that the differentiation between close and remote protons is due to the isotopic gradient defined by the molecular surface. In fact, this helps. But the same proton spin diffusion barrier is still visible in the absence of deuteration [29].

At this point, the evolution of the proton spin polarisation is described by two steps

1. Polarisation of the close protons in $R_{1}$. The 20 protons of EHBA-Cr(V) are in contact with the electron–electron spin exchange reservoir $R_{0}$. They give rise to the intensity of polarised neutron scattering.
2. Transfer of the polarisation of the close protons in $R_{1}$ to a comparable number of protons in $R_{2}$, the protons of the deuterated solvent. The polarised protons in $R_{2}$ are seen by NMR. Their influence on the intensity of neutron scattering is very small.

**Fig. 6:**
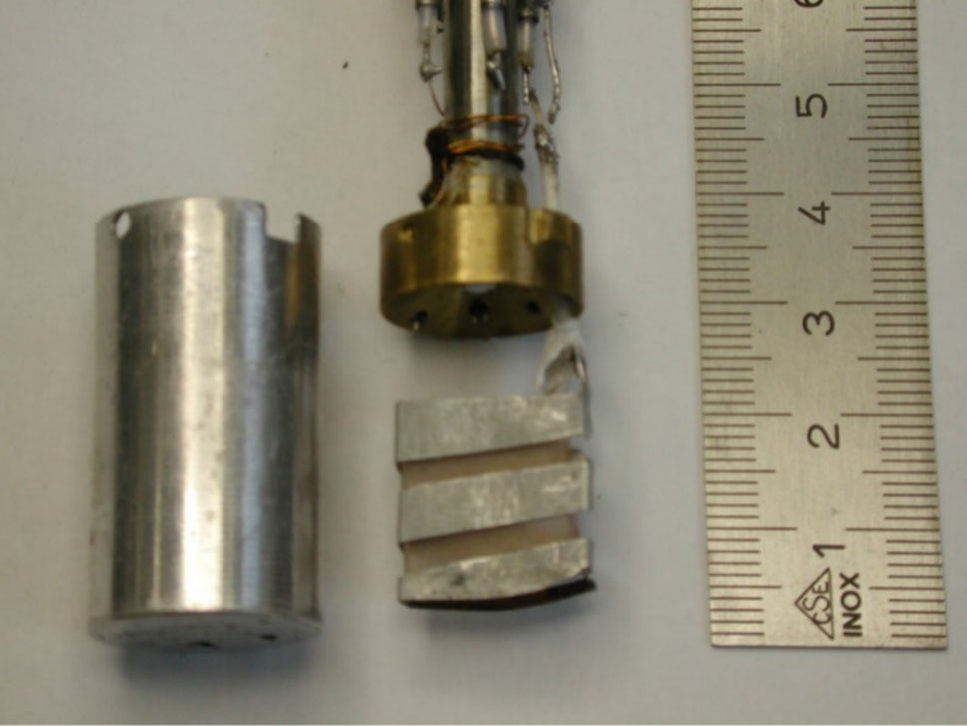
NMR coil, microwave guide and high frequency cavity of the Paul-Scherrer Institute, (PSI) Villigen, Switzerland. All experiments on time-resolved polarised neutron scattering were done with this set-up [28–31]

This experiment has been done using a dynamic polarisation facility operated at a temperature of 1 K in a magnetic field of 3.5 T. It has been developed at the Paul-Scherrer Institute (PSI) at Villigen, Switzerland and modified for neutron scattering experiments (van den Brandt et al. [28]). A 3.5 T split coil wound on an aluminium former is attached to the bottom of a liquid ^4^He vessel (Fig. 6). A $\emptyset$ 49 mm stainless steel tube with an aluminium end cap runs axially through the helium bath and then in vacuum down to the centre of the magnet (Fig. 6). It accommodates a continuous flow ^4^He refrigerator insert with a top loading sample holder device [28–31]. The set up allows a quick sample change.

Most of the time-resolved polarised neutron scattering experiments were done at the small-angle scattering instrument D22 of the Institut Laue–Langevin (ILL) Grenoble. We used polarized incident neutrons of wavelength λ = 4.6 Å with a wavelength spread ∆λ/λ = 0.1 (full width at half maximum). The detector on D22 is a large area single vessel multi-wire proportional counter using ^3^He gas at 1.8 Bar. It contains two orthogonal sets of 128 cathode wires. The position information is extracted by finding a coincidence of an X and Y signal. Such a structure is limited to a counting rate of at most few hundred kilohertz.

### The intramolecular nuclear spin diffusion barrier does it: three reservoirs ${\varvec{R}}_{1}$, ${\varvec{R}}_{2}$, and ${\varvec{R}}_{3}$

**Fig. 7:**
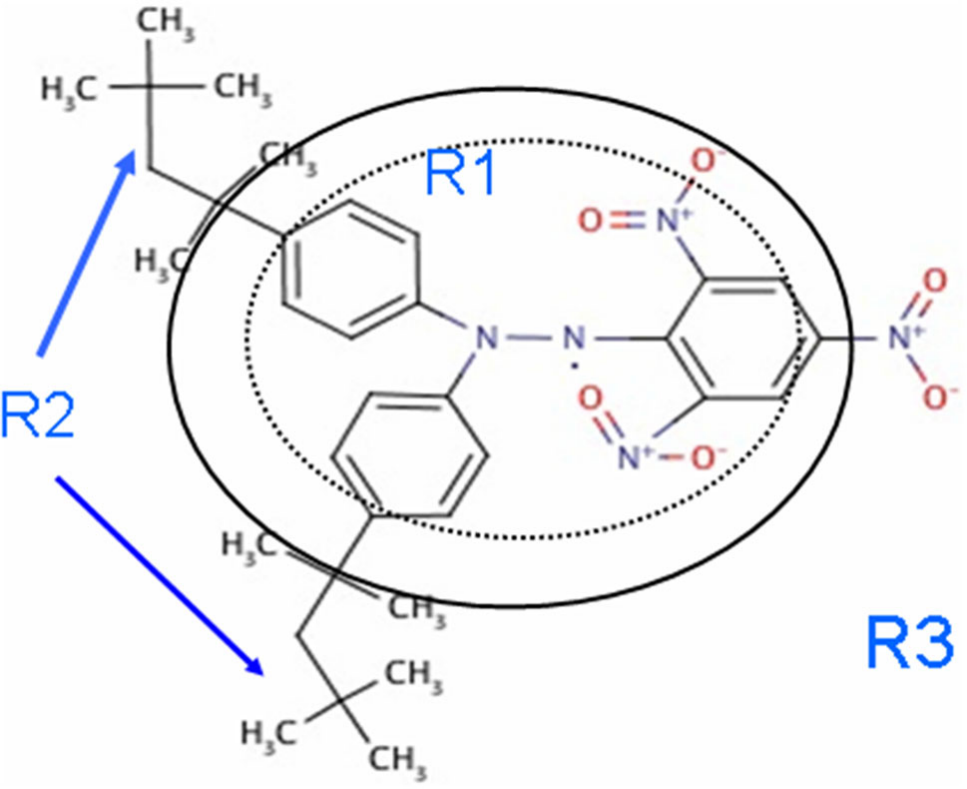
2,2-di(tert-octylphenyl)-1-picryl-hydrazyl free radical. Two octyl groups ($R_{2}$) are separated from the unpaired electron of the picryl-hydrazyl by phenyls. The electron spin density (dotted line) is known from magnetic neutron diffraction [32]. $R_{3}$ = solvent. The radius of $R_{1}$ is 7 Å (solid line)

Thanks to the initiative of Ben van den Brandt (PSI), medium size free radicals were studied by time-resolved polarised neutron scattering. These were supposed to be large enough to show an intramolecular proton spin diffusion barrier. The free radical 2,2-di(tert-octylphenyl)-1-picryl-hydrazyl appeared to be promising because of its two extended octyl groups. The electron spin density of this free radical is known from magnetic neutron diffraction [32]. It includes the phenyl groups of DPPH (dotted line in Fig. 7). The magnetic spin diffusion barrier extends beyond the region of significant electron spin density by about 2 Å at most (line in Fig. 7) [29].

**Fig. 8:**
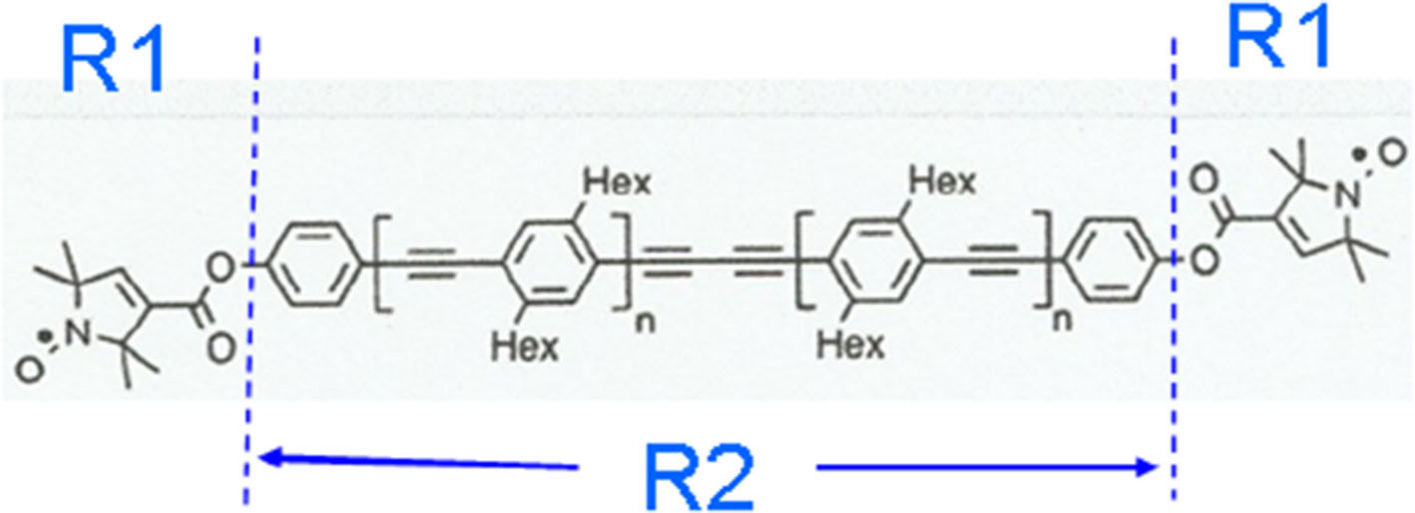
A biradical (n = 1). The distance between the unpaired electrons is 40 Å. The reservoir $R_{2}$ contains four hexyl groups, C_6_ H_13_. The protons of the deuterated solvent constitute $R_{3}$. The radius of $R_{1}$ is 5 Å. The biradical sample is courtesy of A. Godt [33]

The molecular structure of the rigid nitroxide biradical is shown in Fig. 8. The best agreement of the measured intensity of time-resolved polarised neutron scattering calculated from its model is obtained by using spherical spin diffusion barriers with a radius of about 5 Å centred at the > N–O· groups of each of the pyrroline rings. Each of the two sub-reservoirs $R_{1}$ contains 13 hydrogen atoms of the pyrroline ring. The four C_6_ H_13_ groups with their 52 hydrogens are outside the magnetic spin diffusion barrier and constitute $R_{2}$ as shown in Fig. 8. The hydrogen atoms of the deuterated matrix belong to $R_{3}$.

The introduction of an intramolecular nuclear spin diffusion barrier requires an extension of the present system by an additional reservoir $R_{3}$. The evolution of proton spin polarization then is described by three steps [29].

1. Polarisation P_1_(t) of the close protons in $R_{1}$. The close protons are in contact with the electron–electron spin exchange reservoir $R_{0}$.
2. Transfer of the polarisation of the close protons in $R_{1}$ to the protons in $R_{2}$ of the solute. The evolution of the proton polarisation P_2_(t) in $R_{2}$ is controlled by the magnetic nuclear spin diffusion barrier. The barrier separating $R_{1}$ from $R_{2}$ is part of the solute.
3. The protons of the deuterated solvent constitute $R_{3}$. Their influence on the intensity of neutron scattering is very small. NMR is sensitive to polarisation in $R_{2}$ and $R_{3}$.

**Fig. 9:**
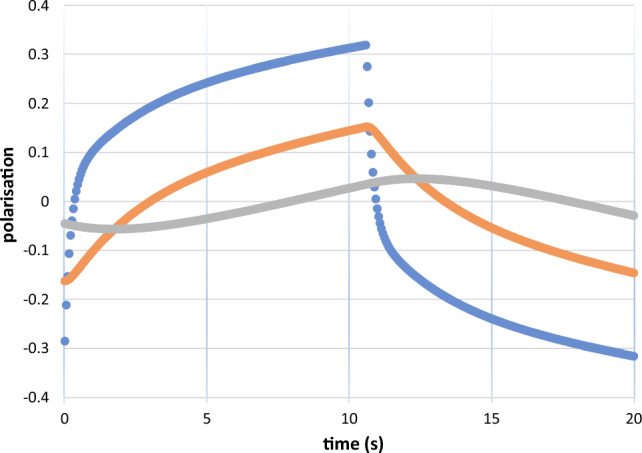
Time-resolved polarised neutron scattering from dynamic polarised protons of DPPH in a deuterated matrix. $$P_{1} \left( t \right)$$$$P_{2} \left( t \right)$$$$P_{3} \left( t \right).$$ Cycle of 20 s

**Fig. 10:**
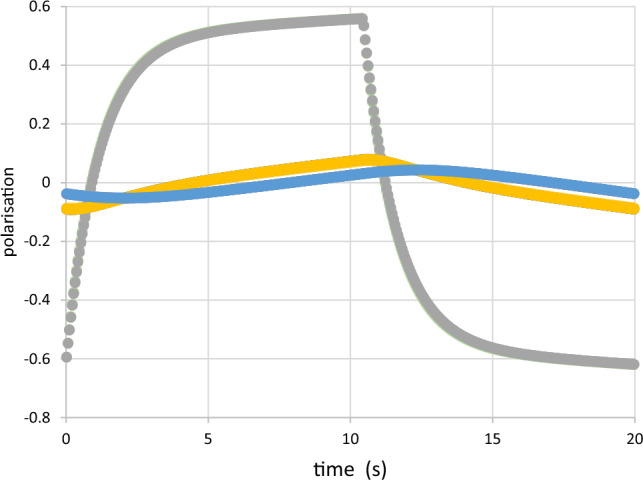
Time-resolved polarised neutron scattering from dynamic polarised protons of the biradical in a deuterated matrix. $$P_{1} \left( t \right)$$$$P_{2} \left( t \right)$$$$P_{3} \left( t \right).$$ Cycle of 20 s

There is a large change of the polarisation $$P_{1} \left( t \right) $$ of the close protons in $$R_{1}$$ after each reversal of the direction of DNP (Figs. 9, 10). The moderate polarisation of the octyl protons of $$R_{2}$$ of DPPH contrast with the very weak polarisation of the hexyl protons of the biradical. The propagation of polarisation along the rigid chain of the biradical to its hexyl groups is strongly impeded. The polarisation of the protons in $$R_{2} $$ of the biradical is close to that of the solvent. In this case, a by-pass from $$R_{1}$$ to $$R_{3} $$ is probably not negligible.

#### Mathematical formalism

Three rate equations govern the flow of nuclear polarisation between the four reservoirs $R_{0}$–$R_{3}$ coupled in series [30, 31].

4 $$ \begin{aligned} & \frac{{dP_{1} }}{dt}\,\, = \,\,\frac{{W_{01} }}{{N_{1} }}\,\left( {P_{0} - P_{1} } \right)\, + \,\frac{{W_{12} }}{{N_{1} }}\left( {P_{2} - P_{1} } \right)\,\, \\ & \frac{{dP_{2} }}{dt}\,\, = \,\frac{{W_{12} }}{{N_{2} }}\left( {P_{1} - P_{2} } \right)\, + \,\frac{{W_{23} }}{{N_{2} }}\left( {P_{3} - P_{2} } \right) \\ & \frac{{dP_{3} }}{dt}\,\, = \,\,\frac{{W_{23} }}{{N_{3} }}\left( {P_{2} - P_{3} } \right) \\ \end{aligned} $$

There are $N_{i}$ protons with the polarisation $P_{i}$ in the reservoir R_i_. $\left| {P_{0} } \right|$ close to 1, is the polarisation of the electron–electron-spin exchange reservoir of $R_{0}$ in contact (or *‘in speaking terms’*) with the close protons. The direction of DNP depends on the sign of $P_{0}$ as it is shown in Fig. 4. The rate constants $W_{ij}$ are defined as probabilities of a mutual spin flip per time unit. The solution of the three coupled linear differential equations in terms of *P*_*1*_*(t)*, *P*_*2*_(*t*) and *P*_*3*_(*t*) is obtained by numerical methods simulating the flow of proton polarisation between the four reservoirs.

Time-resolved polarised neutron scattering is now written as

5 $$ \begin{aligned} & \left( {\frac{d\sigma \left( t \right)}{{d\Omega }}} \right)_{coh.} \, = \, \left| {B_{0} } \right|^{2} + \frac{1}{4} \left| {B\left( t \right)} \right|^{2} \, + \,p {\text{Re}}\left( {B_{0} B^{*} \left( t \right)} \right)\,\,{\text{with}} \\ & B_{o} = \mathop \sum \limits_{n = 1}^{N} b_{n}^{\left( 0 \right)} \exp \left( { - i{\varvec{Q}} \cdot {\varvec{r}}_{n} } \right)\\ &B = \mathop \sum \limits_{j = 1}^{3} P_{j} \left( t \right) \mathop \sum \limits_{n}^{{\left( {N_{j} } \right)}} b_{n} {\text{exp}}\left( { - i{\varvec{Q}} \cdot {\varvec{r}}_{n}^{\left( j \right)} } \right) \\ \end{aligned} $$

${\mathbf{Q}}$ is the momentum transfer. $b_{n} = 1.456 10^{ - 12} \,\, {\text{cm}}$ for polarised protons.

The calculation of the intensity of coherent scattering assumes a more elegant form with the expansion of ${\text{exp}}\left( { - i{\varvec{Q}} \cdot {\varvec{r}}} \right)$ as

6 $$ \begin{aligned} & \exp \left( {i{\varvec{Q}} \cdot {\varvec{r}}} \right) = 4\pi \mathop \sum \limits_{l = 0}^{\infty } \mathop \sum \limits_{m = - l}^{m = l} i^{l} j_{l} \left( {Qr} \right)Y_{l,m}^{*} \left( {\theta ,\phi } \right) Y_{l,m} \left( {\vartheta ,\varphi } \right)\,\,{\text{with}} \\ & {\varvec{Q}} = \left\{ { Q,\,\theta ,\,\phi } \right\} {\varvec{r}} = \left\{ { r,\vartheta ,\varphi } \right\} \\ \end{aligned} $$

Time-resolved polarized neutron scattering then is written as

7 $$ S_{coh} \left( {Q,t} \right) = 2\pi^{2} \mathop \sum \limits_{l = 0}^{L} \mathop \sum \limits_{m = - l}^{l} \left| {B_{l,m}^{\left( 0 \right)} \left( Q \right) + \mathop \sum \limits_{j = 1}^{j = 3} P_{j} \left( t \right) B_{l,m}^{*\left( j \right)} \left( Q \right)} \right|^{2} $$

Equation 7 holds for a completely polarised neutron beam, $\left| p \right|$= 1.

We often refer to the cross term of (7)

8 $$ Z\left( {Q,t} \right) = 4\pi^{2} \mathop \sum \limits_{l = 0}^{L} \mathop \sum \limits_{m = - l}^{m = l} {\text{Re}} \left( {B_{l,m}^{\left( 0 \right)} \left( Q \right) \mathop \sum \limits_{j = 1}^{j = 3} P_{j} \left( t \right) B_{l,m}^{*\left( j \right)} \left( Q \right)} \right) $$

The index L of termination is adapted to the symmetry of the molecular structure. In practice L = 4 may be sufficient. For spherical structures L = 0 will do.

The data of time-resolved neutron scattering from DPPH and from the biradical were analysed in terms of (8) and (9) as it is shown in Figs. 9 and 10. The assumption of a magnetic diffusion barrier is necessary and sufficient to fit the experimental data [29].

The iterative approximation of the experimental data I(Q,t) (see (9) e.g. or Fig. 15) by Z(Q,t) defined by (8) is written in Pascal from Borland Delphi 5 running under Windows 11.

## Tracing the tyrosyl radical in bovine liver catalase

There are currently two methods for varying proton polarisation currently used in time resolved polarised neutron scattering from dynamic polarised proton spin targets

1. The method of proton polarisation direction change. A jump of the time derivative of proton polarisation is achieved by a change of the microwave frequency [34].
2. The method of proton polarisation jump using the NMR method of adiabatic fast passage [26]

The possible merits of the method of the proton polarisation jump by AFP have been alluded to by Buckingham [35].

**Fig. 11:**
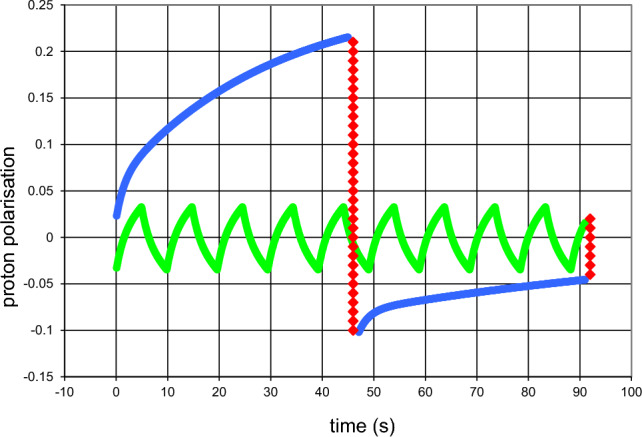
Two methods for varying dynamic proton polarisation. 1. The AFP modulated proton polarisation  with the half cycles of DNP followed by relaxation. At the end of each half-cycle the proton polarisation is reversed to a good extent by the method of adiabatic fast passage, AFP . 2. The other method for varying proton polarisation  consists of two half-cycles as well. There is dynamic proton polarisation (DNP) in the positive direction at a microwave frequency slightly below the EPR followed by DNP in the negative direction at a frequency slightly above the EPR. The polarisation indicated in the figure is that of the protons close to the radical site

The two methods shown in Fig. 11 work on different time scales. A fairly long period is needed for the preparation of a significant jump of the proton polarisation by using AFP (Fig. 11). A rf scan across the NMR profile of about 0.3 s is needed to reverse the proton polarisation.

Alternatively, the direction of dynamic proton polarisation is “switched” by actuating a three-way waveguide structure providing the connection to one or the other source tuned to frequencies above and below the EPR, respectively. The mechanical rotation of the valve by 90° takes 164 ms consisting of 7 ms for closing, 150 ms closed, 7 ms for opening.

### Alternating direction of DNP: four reservoirs R1, R2, R3 and R4

The method of alternating direction of DNP has been used in most experiments of time-resolved polarised neutron scattering from dynamic polarised protons. The results of this method from free radical molecules have been presented in the preceding chapter 4. While the analysis of these experimental data appears to be quite straight forward, those of tyrosyl doped catalase present a major difficulty: the noise. The intensity of the polarisation dependent intensity is low: less than 1:1000 of the total intensity of neutron scattering.

The only thing we know for sure about these data is that the polarisation dependent intensity will assume two extreme values: a minimum and a maximum during one cycle of 10 s. It is for this reason why we preferred a Fourier series truncated at n = 1 for the time dependence of the polarisation dependent intensity I(Q,t) [34].

9 $$ I\left( {Q,t} \right) = a_{0} \left( Q \right) + a_{1} \left( Q \right) cos\left( t \right) + b_{1} \left( Q \right) {\text{sin}}\left( t \right) $$

**Fig. 12:**
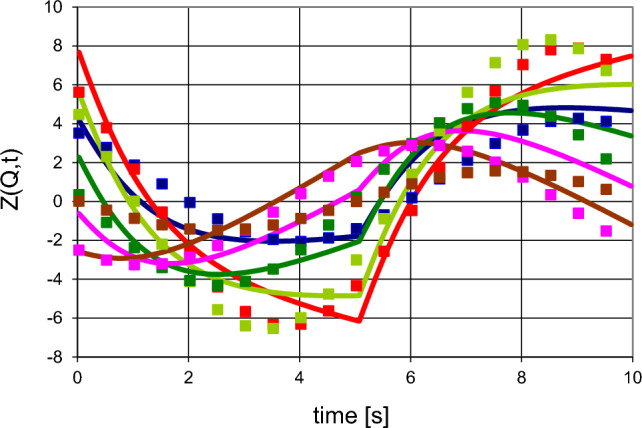
Intensity of polarisation dependent intensity Z(Q,t) versus time at various Q (in Å^−1^).  0.033,  0.044,  0.055,  0.066,  0.077,  0.088, Symbols represent experimental data after two-dimensional Fourier analysis of the experimental data after subtraction of $a_{o} \left( Q \right)$. Lines are Z(Q, t) from the model (after Zimmer et al. [34])

Once the set of reduced data has been established (Fig. 12), the analysis of the experimental data proceeds in terms of Z(Q,t) as described by (8). The model of the reservoirs to $R_{3}$ proposed in (2) is adapted to the larger structure of catalase. The close protons are confined in a sphere of 1 nm diameter centred at the radical site. A concentric slightly larger sphere of 2 nm diameter defining the new reservoir $R_{2}$ contains not so close protons. The protons outside $R_{1}$ and $R_{2}$ of the catalase molecule constitute $R_{3}$. The residual protons of the deuterated solvent belong to $R_{4}$. The Eqs. 2–8 are adapted to the model enlarged by one additional reservoir [34].

Now let us look for those tyrosine amino acids which may have been converted into a tyrosyl radical. The cross term Z(Q,t) is calculated for each of the 20 tentative radical positions and compared with the reduced experimental data.

**Fig. 13:**
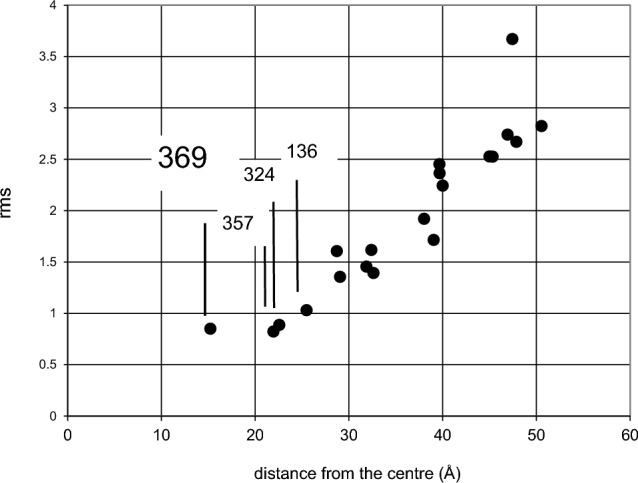
The rms deviation of Z_calc_(Q, t) of the model from Z_exp_(Q, t) of the experimental data for 20 tentative tyrosyl sites (after Zimmer et al. [34]). We used the catalase structure from Fita and Rossman

It appears that there are 4 tyrosine sites which might have become a radical centre: tyr-369, tyr-357, tyr-324 and tyr-326 (Fig. 13). All of them are close to the centre of the tetramer catalase molecule. They are marked in dark blue in the graphical abstract. EPR studies show that tyr-369 did become a radical [22, 23, 36].

The number of polarised protons polarised in excess per tyrosyl is distributed among $$R_{1}$$ and $$R_{2}$$: $$n_{1}$$ = 1.6 in $$R_{1}$$ and $$n_{2} $$ = 0.6 in $$R_{2}$$. The corresponding change in scattering length is 3.2 × 10^−12^ cm. The scattering length of magnetic neutron scattering from an unpaired electron is an order of magnitude smaller [34].

The characteristic time for the build-up of local polarisation in $R_{1}$ is around 1 s. The polarisation of the close protons in $R_{1}$ reaches P = 0.08 after 5 s of microwave irradiation [34].

### The proton polarisation jump using AFP: five reservoirs ${\varvec{R}}_{1}$, ${\varvec{R}}_{2}$, ${\varvec{R}}_{3}$, ${\varvec{R}}_{4}$ and ${\varvec{R}}_{5}$

The analysis of the experimental data from March 2000 presented in this section started in 2017. A draft of the paper entitled “Time-resolved polarised neutron scattering from AFP modulated polarized protons of a free radical and of tyrosyl doped catalase. Tyr-369* confirmed”, by P. Hautle, O. Zimmer, H.M. Jouve, and myself is presently under discussion. In view of the forthcoming publication of this paper we restrict its presentation in this Festschrift to a brief account which allows a guess about future experiments on polarized neutron scattering on AFP modulated targets.

Time-resolved neutron scattering from tyrosyl doped catalase has been measured at 6 microwave frequencies close to the EPR profile of the tyrosyl. i.e.

**Fig. 14:**
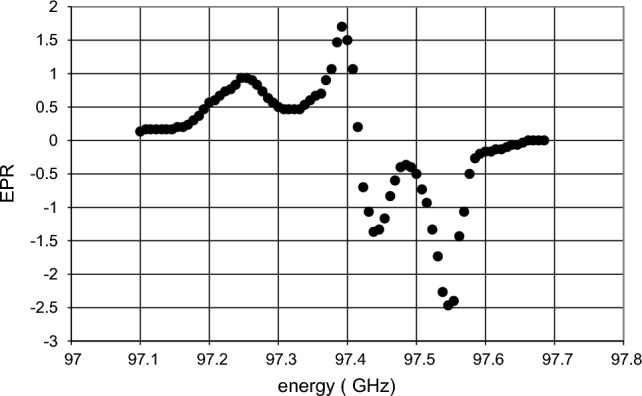
The EPR spectrum of the tyrosyl radical (●●●●●), after Ivancic et al. [22]. The EPR spectrum is defined by the hyperfine interaction of the unpaired electron with the four ring protons of the phenyl group and the β protons of the methylene group. The hyperfine interactions with the β protons depend on their orientation with respect to the phenyl group [36]. The EPR spectrum was obtained under non-saturating conditions. Temperature 10 K, microwave frequency 244.996 GHz, modulation amplitude 10 G (at 100 kHz) modulation frequency 10 kHz [22]

1. Below the EPR of tyrosyl: 97.15 GHz, 97.20 GHz, and 97.25 GHz
2. Above the EPR of tyrosyl: 97.45 GHz, 97.5 GHz, and 97.55 GHz

and a seventh one at E = 96.8 GHz assumed to be off-resonance (Fig. 14).

The direction of DNP is expected to be positive at microwave frequencies below the EPR of tyrosyl and it will negative at microwave frequencies above the EPR. The protocol of time-resolved neutron scattering is shown in Fig. 11. At each energy the cycle was repeated 140 times.

The polarization dependent intensity of neutron scattering from tyrosyl doped catalase is weak. This is true for both the time resolved neutron scattering from a sample subjected to alternating directions of DNP (5.1) and targets the polarisation of which is modulated by AFP (5.2). While in the first case a reduction of the raw data to a truncated Fourier series turned out to be useful [34], a similar procedure cannot be applied to an evolution of the neutron scattering intensity, periodically discontinued by the application of AFP.

The periodic reversion of the proton polarisation each 46 s requires a separate description of each of the two half-cycles. The variation of the scattering intensity with time is approximated by a first order polynomial, which is assumed to hold for most of the time after each application of AFP. In a next step the variation of the parameters of the straight lines with the momentum transfer is smoothed by a moving average. Needless to say, that this step bears uncertainties which enter into the analysis using Eq. 9. They will be discussed in the forthcoming paper.

**Fig. 15:**
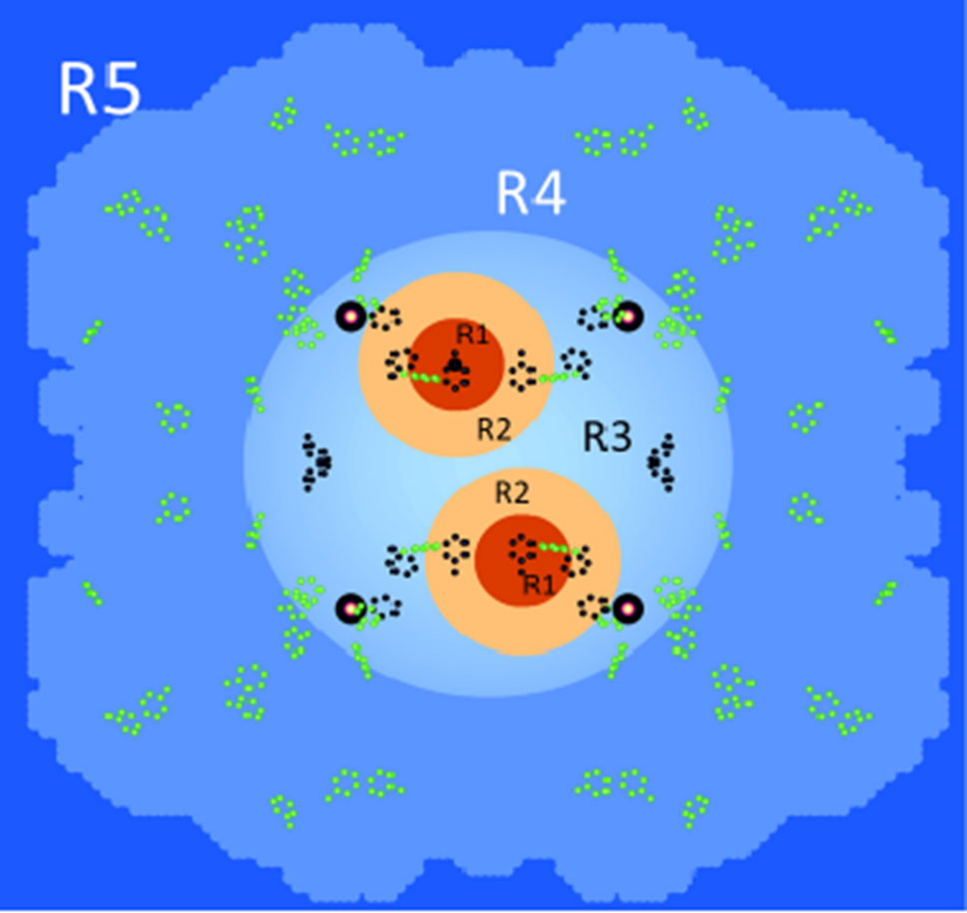
Creation of proton polarisation at the tyrosyl sites and its propagation into the bulk. The sources of DNP are shown as red spheres of $R_{1}$ surrounded by shells of not so close protons of $R_{2}$ (in o orange). The region $R_{3}$ includes the four heme groups shown by their iron atoms (black circle). The regions preferentially affected by AFP are in blue. The efficiency of AFP increases with the distance from the radical centres. The phenyl group atoms of the tyrosine amino acids are marked by green points. The possible tyrosyls from the method of alternating direction of DNP are shown in dark blue [34]. The site of the tyr-369* radical known from EPR [22] is confirmed by time-resolved neutron scattering using AFP modulated proton polarisation. We used the catalase structure from Fita and Rossman [37]

The analysis of the reduced data in terms of Z(Q,t) defined by (8) starts from the model of catalase shown in Fig. 15.The model of catalase is tentatively enlarged by a further reservoir which separates local magnetic fields in the core region of catalase from the rest of the molecule. Thus, we have, $R_{0}$ and $R_{1}$ as before, $R_{2}$ as defined in 5.1, $R_{3}$ contains the protons of the core of the catalase molecule, those of $R_{1}$ and $R_{2}$ excepted. $R_{4}$ includes the protons of catalase outside $R_{4}$. The protons of the deuterated solvent belong to $R_{5}$. The sites of tyrosyl radicals and the radius of the tentatively introduced reservoir $R_{3}$ are obtained from the data of time-resolved neutron scattering.

With this model in mind, we start the analysis of the reduced data. In a first step, we try to obtain the radius of $R_{3}$ from the reduced data at each microwave energy shown at the top of this section. The transition probabilities $W_{i,j}$ governing the flow of the proton polarisation between the reservoir $R_{1}$–$R_{5}$ are chosen in such a way that Z(Q,t) of the model approaches the polarisation dependent intensity of the reduced data. The best agreement between model and experiment is obtained with a radius of 30 Å for $R_{3}$. A glance at Fig. 15 shows that $R_{3}$ includes the iron atoms of the heme groups of catalase. Moreover, the spherical surface of $R_{3}$ separates the core region of the catalase molecule with a relatively high proton polarisation of 3% from the much less polarised protons of the surrounding $R_{4}$ (lower part of the graphical abstract). The tentative introduction of $R_{3}$ as additional reservoir appears to be justified.

**Fig. 16:**
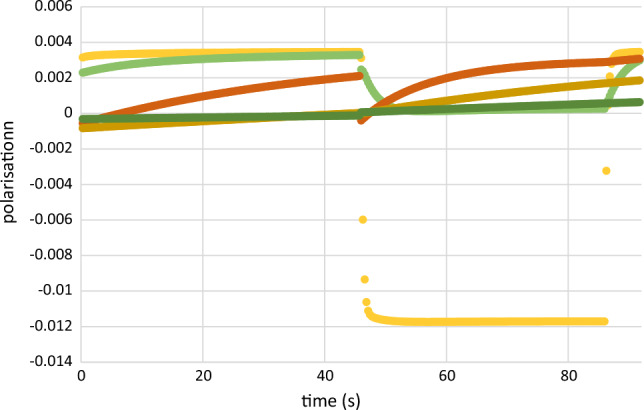
The proton polarisation of the reservoirs obtained from data at E = 97.20 GHz (negative direction of DNP). $$R_{1}$$, $$R_{2}$$, $$R_{3}$$, $$R_{4}$$, $$ R_{5}$$. At this microwave frequency the evolution of the proton polarization with time is almost entirely governed by the drift of proton polarization to its value at thermal equilibrium, that is periodically interrupted by its AFP induced reversal

The diagrams showing the evolution of the reservoir dependent evolution of the proton polarisation vary with the microwave frequency in a plausible way. There is one exception which comes as a real surprise. The dynamic polarisation of the protons into the negative direction at E = 97.5 GHz is very weak, contrary to that of the neighbouring energies E = 97.45 and 97.55 GHz. We may consider this is a signature of the EPR line shown in Fig. 14. In the absence of a significant flow of the negative polarisation emerging at $R_{1}$, there will be a drift of the proton polarisation towards its value $P_{e}$ = 0.35% at thermal equilibrium. We are left with an interplay of AFP and spin lattice relaxation (Fig. 16).

NMR techniques like AFP in experiments of polarised neutron scattering from dynamic polarised targets clean up the proton polarisation of the target. The aim is to render some parts of a macromolecular structure more visible to polarised neutron scattering. The way how this may work is shown on the right side of the graphical abstract. On the top of this column, we have the dynamic polarisation of the protons close to the radical site, The spreads out into the bulk by passing through a sequence of reservoirs $R_{2}$–$R_{5}$. The efficiency of AFP is highest with the most remote protons in $R_{5}$ (shown in dark blue). It is lower with the regions closer to the source of DNP. Only 6% of the close protons in $R_{1}$ may change their direction of polarisation during a rf scan across the NMR profile [38]. It is the complementarity of these two mutually opposite actions which allows one to tailor the spatial distribution of proton polarisation to the aims of the experimentalist.

The flow of proton polarisation in hydrogenous materials may primarily be influenced by two kinds of gradients:

1. Isotopic composition
2. Local magnetic field

The isotopic composition may change at the surface of a protein rich in protons dissolved in a deuterated solvent. An isotopic gradient has been shown to be a most efficient proton spin diffusion barrier [28].

The detection of a magnetic inhomogeneity by time-resolved polarised neutron scattering from a dynamic polarised target has been touched here only briefly. It is subject of a forthcoming paper by Hautle et al. mentioned above.

## Conclusion

This article has been written with the intention to throw light on 50 years development neutron small angle scattering at the ILL, with D11 as flagship and later enriched by D22. We gratefully remember Konrad Ibel and Roland May to whom we owe the success of these instruments.

But off the main track relying on the substitution of hydrogen isotopes, something less known but intimately related to neutron scattering developed for a long time outside the ILL, the technique of polarised neutron scattering from dynamic polarised targets. At its final stage, it needed the power of D22 of the ILL.

Nuclear dynamic polarisation is a powerful method which may reach complete polarisation as it has been shown for protons [7]. The advantages of neutron scattering from polarized nuclei are.

1. Low incoherent scattering with positive DNP
2. Huge change of coherent scattering intensity

Both aspects are and will remain important in structural studies on hydrogenous material. Proton spin targets and deuteron spin targets, obtained after selective depolarisation of one isotope species, open new horizons in contrast variation. The polarizing agent is a radical which has been added to the sample. As such it is exogenic source of polarisation, unlike the tyrosyl radical.

In life sciences we know about the extraordinary capabilities of radicals which are part of enzymes. These radicals are endogenous. The way how they work often remains a mystery. The reaction they facilitate may be so fast that individual chemical steps may remain undetected. With catalase we have presented an example of a suicide substrate which interacts with the enzyme much more slowly leading to the conversion of one of its amino acids into a radical.

Whatever the nature of the radicalized macromolecular structure is, an answer to the following two questions is within reach.

1. Where are the radical sites?
2. How does the proton polarisation emerging at the radical site spread out?

It appears that time resolved polarised neutron scattering from AFP modulated polarisation of protons inside a macromolecule provides a more precise localisation of radical sites. Magnetic inhomogeneities, that are not supporting DNP and therefore considered as passive elements, then show up more clearly.

With further advance in polarised neutron scattering from dynamic polarised protons, we hope to trace the path of an unpaired electron from the site of its creation to the final destination. Time-resolved polarised neutron scattering in combination with pulsed microwaves might be promising.

## Data Availability

No Data associated in the manuscript.
